# Comparative Efficacy and Cost-Effectiveness of Two-Layer Versus Four-Layer Compression Bandages for Venous Leg Ulcers: A Prospective Study

**DOI:** 10.7759/cureus.68189

**Published:** 2024-08-30

**Authors:** Bhushan Shah, Adithya R Vijendra, Jayant Bajaj

**Affiliations:** 1 General Surgery, Dr. D. Y. Patil Medical College, Hospital & Research Centre, Dr. D. Y. Patil Vidyapeeth (Deemed to be University), Pune, IND

**Keywords:** two-layer bandaging, four-layer bandaging, cost-effectiveness, chronic venous insufficiency (cvi), compression therapy, prospective study, ulcer treatment, venous leg ulcers, ­wound healing

## Abstract

Objective: This study aims to evaluate and compare the efficacy and cost-effectiveness of two-layer versus four-layer compression bandages in the treatment of venous leg ulcers (VLUs).

Methods: A prospective study was conducted at a tertiary hospital from August 2022 to July 2024. A total of 100 patients with chronic VLUs were sampled. Of the patients, 50 were given two-layer (group A) compression therapy, and the other 50 were given four-layer (group B) compression therapy. Outcomes after both therapies were analyzed.

Results: The mean age of the participants was 45.76 years, with a predominance of males (67%). Both bandaging systems demonstrated similar healing efficacy with no significant difference in ulcer size or healing time between groups. However, the four-layer bandage system required significantly fewer follow-ups (mean = 4.88) compared to the two-layer system (mean = 6.46) (p < 0.001). The mean total cost was higher for the four-layer system (₹3416) compared to the two-layer system (₹2907) (p = 0.004). Complications such as pain and pressure ulcers were comparable, though the four-layer system was associated with slightly higher discomfort and skin irritation.

Conclusion: The four-layer bandage system may offer marginal advantages in wound healing and fewer follow-ups, but it is more expensive. The two-layer bandage system is more cost-effective and patient-friendly.

## Introduction

Chronic venous insufficiency is a debilitating disease that more commonly affects women. A serious complication of chronic venous insufficiency is the development of venous leg ulcers [1]. The majority of ulcers that appear in outpatient departments are venous ulcers. This condition has a high rate of morbidity, is challenging to treat, and is very expensive for patients and the healthcare system. Venous ulcers are a well-known medical problem that is nevertheless inadequately handled, even though wound care clinics were created as early as 1500 BC and have been for more than 3.5 millennia [2]. This is true even though our knowledge of the etiology and management of venous ulcerations has advanced significantly. This is despite significant advancements in understanding the pathogenesis and treatment of venous ulcerations. It is often observed that a lot of doctors prefer to treat the wound itself instead of dealing with the root cause of the issue [2].

The pathophysiology of venous leg ulcers starts with the development of ambulatory venous hypertension [3]. Although ulcers can occur spontaneously, trauma is the most common trigger for the development of ulcers over pre-ulcerative lesions. This trauma is often minor and may go unnoticed by the patient [4]. Venous ulcers present a serious dilemma for treating physicians and impose an economic burden on healthcare services. With an increasing number of patients suffering from venous leg ulcers, it is vital to develop cost-effective treatment strategies, as treatment is often expensive, long-term, and has a high risk of recurrence [5].

Leg ulcers are thought to affect 1% of people in developed nations, with a 0.1%-0.2% prevalence [6]. Skin pigmentation, lipodermatosclerosis, and ulceration are linked to the ensuing venous hypertension and compression therapy is generally acknowledged as the mainstay of care [7]. Other specific medications that claim to promote ulcer healing are of questionable efficacy, and antibiotics do not accelerate ulcer healing in the absence of cellulitis. Lowering the vein diameter in the superficial and deep systems is possible with appropriate compression, albeit not all patients experience this. A soft roll, crepe bandage, elastic bandage, and a final cohesive holding layer make up the four-layer bandage system. On the other hand, the two-layer method comprises a crepe bandage and a soft roll.

In the present study, we aim to evaluate the efficacy of a two-layer compression bandage compared to a four-layer compression bandage in the healing of venous leg ulcers.

## Materials and methods

This prospective study was conducted at a tertiary hospital from 1st August 2022 to 31st July 2024. Ethical approval for this study was obtained from the Institutional Review Board - Ethics Committee, Dr. D. Y. Patil Vidyapeeth, Pune (Approval No.: IESC/PGS/2022/81). All participants provided written informed consent prior to enrolment in the study. The primary aim was to compare the efficacy and cost-effectiveness of two-layer versus four-layer compression bandages in the treatment of venous leg ulcers.

Out of 250 patients who presented to the outpatient department, 100 patients were selected from age 18 years and older, with venous ulcers in the lower limb that were at least one month old and measured between 5 cm² and 25 cm² in area. Patients who were excluded had concurrent peripheral vascular disease, an ankle-brachial pressure index (ABPI) greater than 1.3 or less than 0.8, active infections requiring surgical debridement, deep venous thrombosis (DVT), congestive cardiac failure, inferior vena cava (IVC) obstruction, or venous ulcers due to vasculitis. Also, patients who did not comply with the treatment protocols or follow-up schedules were excluded. They were randomly allocated into two groups: 50 patients in group A received two-layer compression therapy, while 50 patients in group B received four-layer compression therapy. Dressings were applied by trained surgery residents or nursing staff. The materials used for the bandages included soft roll, crepe bandage, elastic bandage, and transpore tape.

Subjects were analyzed on the basis of age, sex, body mass index (BMI), ABPI, and Doppler findings, but the primary outcome measure was the size of the ulcer, which was recorded at the initial presentation and at each subsequent dressing change to monitor healing progress. Complications following compression therapy, such as pain, pressure sores, and contact dermatitis, were thoroughly inspected, and patients were asked about any discomfort, pain, or joint stiffness. The cost of treatment was calculated based on the cost per dressing (450 rupees for two layers and 750 rupees for four layers) and the total number of weeks required for complete healing.

With the help of SPSS version 20 (IBM Corp., Armonk, NY), data were input into Microsoft Excel (Microsoft Corporation, Redmond, WA) and examined. Descriptive statistics, such as mean, median, standard deviation, and interquartile range for continuous variables and frequencies and percentages for categorical variables, were used to examine baseline demographics and clinical data. Using parametric and non-parametric tests based on normality testing, such as chi-square tests for categorical variables, t-tests and ANOVA for continuous variables, and Cohen's test to measure the effect for indicating the standardized difference between two means, the inferential statistical analyses were carried out. A p-value less than 0.05 was the threshold for statistical significance.

## Results

The analysis of the patient population prior to initiating treatment for venous ulcers showed that participant ages ranged from 18 to 82 years, with the majority (40 out of 100) being in the 31 to 45-year age group and predominantly male (67 out of 100). The participants' BMI varied from 18.5 to 36, with an average of 27.56. The mean ABPI was 1.15 for the four-layer group and 1.12 for the two-layer group. The average ulcer size was 9.04 cm² in the four-layer group and 9.13 cm² in the two-layer group.

The four-layer group required significantly fewer follow-ups (mean = 4.88) compared to the two-layer group (mean = 6.46), with a p-value of less than 0.001 and a Cohen's d of 1.03, indicating a large effect size (Tables 1, 2).

**Table 1:** Comparison of follow-ups needed between the two groups (N = 100).

		N	Mean	Standard deviation	Standard error mean
Number of follow-ups needed	4-layer	50	4.88	1.38	0.2
	2-layer	50	6.46	1.68	0.24

**Table 2:** The number of follow-up appointments required was analyzed using various statistical tests. Data are expressed in frequency, mean ± SD, and p-value by chi-square test. df: degrees of freedom.

	t-value	df	p-value	Cohen's d
Equal variances	-5.14	98	<0.001	1.03
Unequal variances	-5.14	94.42	<0.001	1.03

Doppler findings showed no significant difference between the two groups (p = 0.184). The distribution of findings across different categories was similar in both groups (Tables 3, 4).

**Table 3:** Doppler findings and type of dressing used. SFJ: saphenofemoral junction; GSV: great saphenous vein; SSV: small saphenous vein.

		Type of dressing used	
		4-layer	2-layer
Doppler findings	SFJ competent, multiple incompetent perforators along GSV, venous reflux > 5 seconds	12	20
	SFJ competent, multiple incompetent perforators along SSV, venous reflux > 5 seconds	21	19
	SFJ incompetent, multiple incompetent perforators, venous reflux > 5 seconds	17	11
	Total	50	50

**Table 4:** Doppler findings in both groups were analyzed using various statistical tests. Data are expressed in frequency, mean ± SD, and p-value by chi-square test. df: degrees of freedom.

	Chi^2^	df	p-value
Doppler findings - type of dressing used	3.39	2	0.184

The distribution of complications (pain, pressure ulcer, contact dermatitis) was not significantly different between the two groups (p = 0.356). Most patients in both groups experienced no complications (Tables 5, 6).

**Table 5:** Comparison of complications with the type of dressing used.

Type of dressing used		Complications				Total
		None	Pain	Pressure ulcer	Contact dermatitis	
	4-layer	36	9	4	1	50
	2-layer	37	12	1	0	50
	Total	73	21	5	1	100

**Table 6:** Complications in both groups were analyzed using various statistical tests. Data are expressed in frequency, mean ± SD, and p-value by chi-square test. df - degrees of freedom.

	Chi^2^	df	p-value
Type of dressing used - complications	3.24	3	0.356

The mean total cost of treatment was significantly higher for the four-layer group (mean = 3416 rupees) compared to the two-layer group (mean = 2907 rupees), with a p-value of 0.004 and a Cohen's d of 0.59, indicating a moderate effect size (Tables 7, 8).

**Table 7:** Comparison of total cost of the procedure between the two groups.

		Total	Mean	Standard deviation	Standard error mean
Total cost	4-layer	50	3416	965.82	136.59
	2-layer	50	2907	756.33	106.96

**Table 8:** The total cost of treatment in both groups was analyzed using various statistical tests. Data are expressed in frequency, mean ± SD, and p-value by chi-square test. df: degrees of freedom.

	t-value	df	p-value	Cohen's d
Equal variances	2.93	98	0.004	0.59
Unequal variances	2.93	92.67	0.004	0.59

## Discussion

Our study showed a male predominance, accounting for 67% of the participants, while females accounted for 33%. This finding aligns with other studies from India, such as those by Alamelu [8] and Kota et al. [9]. However, our results contrast with the studies by Gethin et al. [10] and Probst et al. [11], who found a higher prevalence of venous ulcers in women. The variation in these findings could be attributed to the smaller sample size of our research as well as the social stigma that Indian women may face in seeking prompt medical attention.

Upon data analysis for the age group, the majority were aged 31 to 45 years (42%). This finding can be compared to a study conducted by J. Bradford Rice [12]. However, a study by Berenguer Pérez et al. [13] stated that the incidence of venous leg ulcers (VLU) increases after the age of 65 years. This discrepancy could be due to the fact that 31 to 55-year-old people in our country are the working class group, which makes them the most common age group affected due to their long hours of travel, work, and the need to provide for family thus neglecting their own health.

In the subgroup analysis of the total number of follow-ups needed for both patient groups, we found that among patients with a four-layer bandage for VLU, the majority of patients required more than four weeks of follow-up. However, all patients with a two-layer bandage needed more than four weeks of follow-up for the ulcer to heal completely. These data were compared with a study by Moffatt et al. [14] and O’Meara et al. [15]. Conversely, Franks et al. [16] stated no significant difference in healing based on the bandaging technique used. Therefore, we conclude that the number of follow-ups a patient needs after four-layer bandaging for venous leg ulcers is significantly less than that for two-layer bandaging.

One of the most important factors when conducting any study is the cost-effectiveness of the new procedure to ensure it is acceptable to a large population at the end of the study. Data revealed that the average cost of four-layer bandaging was significantly more than two-layer bandaging. These data align with a study performed by O'Brien et al. [17]. Thus, we inferred that the cost of four-layer bandaging was notably higher than that of two-layer bandaging.

In our study, we found that the majority of the patients experienced no complications from either procedure. However, more patients with two-layer bandaging than four-layer bandaging reported pain during their follow-up visits. Additionally, when evaluating other complications such as pressure ulcers and contact dermatitis, we found that four and one out of 50 patients with four-layer bandaging developed pressure ulcers and contact dermatitis, respectively, while no patients with two-layer bandaging developed contact dermatitis. Accordingly, we can conclude that the risk of developing complications like pain and pressure ulcers is significantly lower with four-layer bandaging compared to two-layer bandaging, although contact dermatitis remains a complication primarily associated with four-layer bandaging. These data regarding complications post-application of four-layer or two-layer bandages for venous leg ulcers were compared with studies by Moffatt et al. [14] and O'Meara et al. [15], which suggested that there is no statistical difference in the development of complications after using either procedure for venous leg ulcer healing.

Limitations of the study

Our study had several limitations. First, a longer observation period would be necessary to better understand the long-term outcomes and potential recurrence of venous leg ulcers following the treatments. Second, an intention-to-treat analysis was not utilized due to the loss of follow-up for many patients, which may have introduced bias and affected the reliability of our results. Additionally, the uniform application of bandaging techniques was compromised due to the unavailability of trained professionals, potentially impacting the consistency and efficacy of the treatments. Lastly, the recurrence of ulcers was not assessed in this study, which is a crucial aspect of understanding the long-term effectiveness and sustainability of the treatments. Further studies are needed to address these limitations and provide a more comprehensive evaluation of the interventions.

## Conclusions

This study highlights the importance of a balanced approach to treating venous leg ulcers, considering both efficacy and cost-effectiveness. The preference for a more economical and patient-friendly two-layer system challenges the prevailing notion that more complex treatments are always superior. Emphasizing patient comfort and cost-efficiency can reshape current treatment paradigms, leading to more sustainable and widely applicable healthcare solutions. By integrating these findings into clinical practice and policy, the overall management of venous leg ulcers can be significantly improved, benefiting patients and healthcare systems alike.
